# MeCP2 post-translational modifications: a mechanism to control its involvement in synaptic plasticity and homeostasis?

**DOI:** 10.3389/fncel.2014.00236

**Published:** 2014-08-13

**Authors:** Elisa Bellini, Giulio Pavesi, Isabella Barbiero, Anna Bergo, Chetan Chandola, Mohammad S. Nawaz, Laura Rusconi, Gilda Stefanelli, Marta Strollo, Maria M. Valente, Charlotte Kilstrup-Nielsen, Nicoletta Landsberger

**Affiliations:** ^1^Division of Neuroscience, San Raffaele Rett Research Center, San Raffaele Scientific InstituteMilan, Italy; ^2^Department of Biosciences, University of MilanMilan, Italy; ^3^Section of Biomedical Research, Laboratory of Genetic and Epigenetic Control of Gene Expression, Department of Theoretic and Applied Sciences, University of InsubriaBusto Arsizio, Italy

**Keywords:** chromatin, MeCP2, mouse models, phosphorylation, post-translational modifications, Rett syndrome, synaptic plasticity

## Abstract

Although Rett syndrome (RTT) represents one of the most frequent forms of severe intellectual disability in females worldwide, we still have an inadequate knowledge of the many roles played by MeCP2 (whose mutations are responsible for most cases of RTT) and their relevance for RTT pathobiology. Several studies support a role of MeCP2 in the regulation of synaptic plasticity and homeostasis. At the molecular level, MeCP2 is described as a repressor capable of inhibiting gene transcription through chromatin compaction. Indeed, it interacts with several chromatin remodeling factors, such as HDAC-containing complexes and ATRX. Other studies have inferred that MeCP2 functions also as an activator; a role in regulating mRNA splicing and in modulating protein synthesis has also been proposed. Further, MeCP2 avidly binds both 5-methyl- and 5-hydroxymethyl-cytosine. Recent evidence suggests that it is the highly disorganized structure of MeCP2, together with its post-translational modifications (PTMs) that generate and regulate this functional versatility. Indeed, several reports have demonstrated that differential phosphorylation of MeCP2 is a key mechanism by which the methyl binding protein modulates its affinity for its partners, gene expression and cellular adaptations to stimuli and neuronal plasticity. As logic consequence, generation of phospho-defective *Mecp2* knock-in mice has permitted associating alterations in neuronal morphology, circuit formation, and mouse behavioral phenotypes with specific phosphorylation events. MeCP2 undergoes various other PTMs, including acetylation, ubiquitination and sumoylation, whose functional roles remain largely unexplored. These results, together with the genome-wide distribution of MeCP2 and its capability to substitute histone H1, recall the complex regulation of histones and suggest the relevance of quickly gaining a deeper comprehension of MeCP2 PTMs, the respective writers and readers and the consequent functional outcomes.

## Introduction

Rett syndrome (RTT) is a devastating disorder that, because of its incidence, is considered one of the main causes of severe intellectual disability in girls (Percy and Lane, 2005). Typical RTT patients appear to develop normally throughout the first 6–18 months of life when neurological development arrests and a regression phase occurs leading to the loss of previously acquired skills. During and after the regression phase, patients develop a host of typical symptoms including the substitution of purposeful hand use with continuous stereotypic hand movements, loss of language skills, the appearance of autistic features, gait abnormalities, breathing irregularities, seizures, scoliosis, hypotonia, and autonomic dysfunctions (Neul et al., 2010).

Back in 1999, the Methyl-CpG binding Protein 2 (*MECP2)* gene was discovered as the genetic cause of Rett syndrome (Amir et al., 1999); since then, hundreds of different mutations of the gene have been associated with RTT and less frequently with other forms of intellectual disabilities, such as autism, schizophrenia, mental retardation and Angelman-like syndrome (Chahrour and Zoghbi, 2007). Lately, duplication and triplication of the gene have also been identified as the genetic cause of the recently classified *MECP2* duplication syndrome that usually affects boys (Van Esch, 2011). The production of several mouse models, carrying different *Mecp2* alterations and phenotypically copying many typical features of the human disease, has indeed provided the formal genetic proof of the involvement of *MECP2* in RTT (Ricceri et al., 2008). Importantly, mouse models of *Mecp2* functions have also permitted to demonstrate that phenotypic rescue is possible at least in mice, suggesting that the *MECP2*-related conditions might be reversible (Guy et al., 2007). These studies have dramatically boosted the research of MeCP2 functions, and have yielded a wealth of evidence proving that MeCP2 functions are required for the maturation and maintenance of proper dendritic arborization and spine formation. Thus, RTT pathogenic mechanisms appear to converge at the synaptic level, disrupting synaptic transmission and plasticity.

At a molecular level, there exist more contradictory data. In fact, MeCP2 appears as a multifunctional protein, mainly but not exclusively involved in regulating gene expression. It appears that the structure of MeCP2 together with a series of differential post-translational modifications (PTMs) might justify this functional versatility, possibly occurring through the capacity of the methyl-binding protein to interact with several diverse protein partners (Klose and Bird, 2004). Thus, in this review we will briefly summarize the animal models of *Mecp2* that have been instrumental for studying Rett syndrome. We will then describe relevant evidence substantiating the functional importance of MeCP2 in synaptic and neuronal plasticity regulation. Since we are certain that the development of well-targeted therapies requires a better comprehension of the functional role(s) of MeCP2, their regulation and their relevance in the pathobiology of RTT, we will analyze in depth the current knowledge of MeCP2 structure and molecular functions and provide bioinformatics and experimental data testifying already established and putative PTMs of MeCP2. Functional studies and animal models used to characterize some of these PTMs will be surveyed, highlighting the major weaknesses in the field and which, in our mind, should be the future challenges for a better comprehension of MeCP2 activities.

## *Mecp2* mouse models recapitulate well the human *MECP2*-related pathologies

The generation of several mouse models carrying different *Mecp2* alterations and generally recapitulating many RTT features has provided a major breakthrough for RTT research (Ricceri et al., 2008). In particular, the mostly used *Mecp2*-null males (*Mecp2^−/y^*) have no apparent phenotype until 3–8 weeks of age, when they start showing gross abnormalities, such as locomotor defects, hindlimb clasping, hypotonia, reduced spontaneous movements, tremors, breathing irregularities and often seizures. Symptoms worsen over time, and the animals die within 6–10 weeks of age. Heterozygous female mice (*Mecp2*^−/+^) are viable, fertile and appear normal up to 4–6 months of life, when they start manifesting RTT-like symptoms. Other models with less severe genetic lesions, often mimicking human mutations, have subsequently been generated, and are nicely reviewed in Ricceri et al. (2008). Knock-in mice, used to address the role of specific events of MeCP2 phosphorylation, are discussed in Section MeCP2 Phosphorylation: Where We Stand and Where We Might Go.

The *Mecp2^308/y^* mice, expressing a hypomorphic MeCP2 pathogenic derivative, have also been widely utilized. The mutation causes a deletion of the C-terminal portion, but spares the two most relevant functional domains, i.e., the methyl-binding domain and the transcriptional repression domain (see Section MeCP2 Functions Depend on the Highly Structured Methyl-binding Domain Embedded in a Disorganized Protein). Even though the overall phenotype is milder, the *Mecp2^308/y^* mouse model shares most of the features characterizing the knock-out animals, including learning and memory deficits (Shahbazian et al., 2002).

Conditional knock-out mice have also been generated and characterized, in order to understand better the etiology of RTT and the role of *Mecp2* in discrete brain regions or cell types. As reviewed in Na et al. (2013), the inactivation of *Mecp2* in single brain areas or neuronal subtypes generally leads only to a subset of the typical RTT features. Importantly, despite the fact that all *Mecp2* mutations investigated so far affect brain functions, they are not associated with neuronal loss. Consistently, a major breakthrough in the field came in 2007, when Dr. Bird and his collaborators demonstrated that *Mecp2*-reactivation in symptomatic adult mice (either *Mecp2^−/y^* or *Mecp2*^−/+^) results in a robust rescue of the general conditions of the animals, including survival and breathing, while mobility, clasping, and tremors were less reversed. Altogether, these studies demonstrated that Mecp2-related disorders are reversible and, at least in mice, they can be treated even at late stages of disease progression (Guy et al., 2007). Importantly, these results have been confirmed by several other subsequent studies (Luikenhuis et al., 2004; Giacometti et al., 2007; Jugloff et al., 2008; Garg et al., 2013).

In spite of these recent enormous advances, we highlight that the knowledge regarding the temporal steps through which the consequences of dysfunctional MeCP2 start to manifest is still limited. In fact, most of the studies have been performed in the so-called pre-symptomatic (3–6 weeks of age) or symptomatic (adult) animals. However, recent experimental results have demonstrated that the inactivation of *Mecp2* at different post-natal ages (from late juvenile to adult) always causes the appearance of RTT-like phenotypes and premature death (McGraw et al., 2011; Cheval et al., 2012; Nguyen et al., 2012). These results demonstrate that MeCP2 functions are essential to maintain neurons in a fully functional state. Conversely, it has recently been shown that subtle but consistent impairments are present even at early post-natal stages, when typical symptoms are not yet overt, both in human heterozygous patients and hemizygous null mice. Furthermore, hemizygous *MECP2* male patients display a severe pathological condition as early as at birth (Schüle et al., 2008). Thus, considering that very few studies investigated the possible roles of Mecp2 during embryonic development (Picker et al., 2006; Santos et al., 2007; De Filippis et al., 2010), we underline the necessity to foster the comprehension of MeCP2 functions also during brain development.

## MeCP2 alterations lead to a synaptic phenotype

Once the relevance of MeCP2 had been demonstrated for the central nervous system, it became imperative to define the expression pattern of the protein during brain development and the associated neuro-pathological abnormalities.

Concerning MeCP2 protein levels, it is generally accepted that its expression in brain mirrors neuronal maturation. That is, MeCP2 increases when neurons develop dendritic arbors, project axons and establish connectivity (Kishi and Macklis, 2004; Neul and Zoghbi, 2004). Interestingly, MeCP2 has been shown to be present in an experimental model that prevents synapse formation, albeit at lower level than normal, suggesting that the abundance of MeCP2, rather than its presence, depends on synapse formation (Neul and Zoghbi, 2004).

Altered MeCP2 expression and activity do not affect the gross structure of the brain, and no obvious signs of degeneration, gliosis or inflammation have been reported in RTT patients (Chahrour and Zoghbi, 2007). The most conspicuous morphological abnormalities in post-mortem RTT patients are reduced brain size and weight, with more subtle alterations, such as reduced dendritic arborization, defects in spine density and morphology, and an increase in neuronal packing, in turn leading to augmented cellular density (Bauman et al., 1995; Belichenko et al., 2009b). Abnormalities in the expression of molecules, such as NMDA, AMPA and GABA receptors, that are crucial for both excitatory and inhibitory synaptic transmission, have also been detected (Johnston et al., 2005). Mouse models of RTT have demonstrated similar defects. In particular, two elegant works from Belichenko et al. (2009a,b) have shown that most of the parameters analyzed were altered in dendrites of *Mecp2*-mutant mice of both genders. In particular, and most strikingly, dendrites were swelled, spine density was altered (generally reduced, but increased in few brain areas), and a smaller head and a longer neck characterized the spines. Overlapping results were obtained observing hippocampal neurons of female RTT patients (Chapleau et al., 2009). Furthermore, neurons generated *in vitro* from induced pluripotent stem cells (iPSCs) derived from RTT patients' fibroblasts showed lower dendritic spine density than control neurons (Moutri et al., 2010).

Taken together, all these findings have led to the hypothesis that the neurological deficits of RTT patients arise because of a failure in synaptic and circuit development and/or maintenance. Accordingly, a number of studies have shown that *Mecp2*-null hippocampal slices are characterized by significantly reduced spontaneous excitatory synaptic transmission, deficits in long-term potentiation (LTP), and long-term depression (LTD). Furthermore, 2-photon time lapse imaging has shown that, at the onset of the disease, *Mecp2*-null somatosensory cortices display remarkable alterations in the dynamics of dendritic spines; on the contrary, when maturation of the connectivity is complete, no differences in spine dynamics are evident in *Mecp2*-mutant mice with respect to their wild-type controls (Landi et al., 2011). Accordingly, it has also been demonstrated that dendritic spine density of hippocampal CA1 pyramidal neurons is lower only at postnatal day 7 (P7), while it does not differ at P15 or later, when symptoms are already well-established (Chapleau et al., 2012). These data support a role of Mecp2 during early development of dendritic spines and suggest that, at least in the *Mecp2*-null mouse model used in these studies, compensatory mechanisms that normalize spine density might occur later on in development. However, it is important to observe that a different study, performed using two diverse *Mecp2*-null lines, reported lower dendritic spine density in hippocampal neurons of animals of 3 weeks of age (Belichenko et al., 2009a). We believe that in the future it will be relevant to address these topics using models of the disease, which are less severe and mimick pathogenic human mutations. Furthermore, similar studies should include both genders: in fact, considering that RTT predominantly affects girls, it is reasonable to assume that heterozygous females represent the appropriate genetic mouse model of Rett syndrome, whereas *Mecp2*-null male mice can be considered a suitable model for addressing the biological function of the *Mecp2* gene.

Finally, evidence is accumulating that overexpression of MeCP2 also affects neuronal plasticity: indeed, decreased dendritic branching and spine density, enhanced excitatory synaptic transmission and altered glutamatergic transmission have been associated with mouse models of the so-called *MECP2* duplication syndrome. Enhanced hippocampal LTP responses have been demonstrated in Mecp2 overexpression mouse lines with respect to their littermate controls (Na et al., 2013).

To conclude, a wealth of evidence now substantiates the functional importance of MeCP2 in the regulation of dendritic structure, synaptic plasticity and homeostasis, therefore providing a rationale for the learning and memory deficits that are constitutively seen in RTT patients and in the mouse models of the disease.

## MeCP2 functions depend on the highly structured methyl-binding domain embedded in a disorganized protein

Starting from the previous observations, we believe that the development of clinical applications implies the urgency of improving our understanding of the functional roles of MeCP2, their regulation, and the protein domains involved. Thus, we will now review the state of art of MeCP2 structure and function.

**Figure 1:**
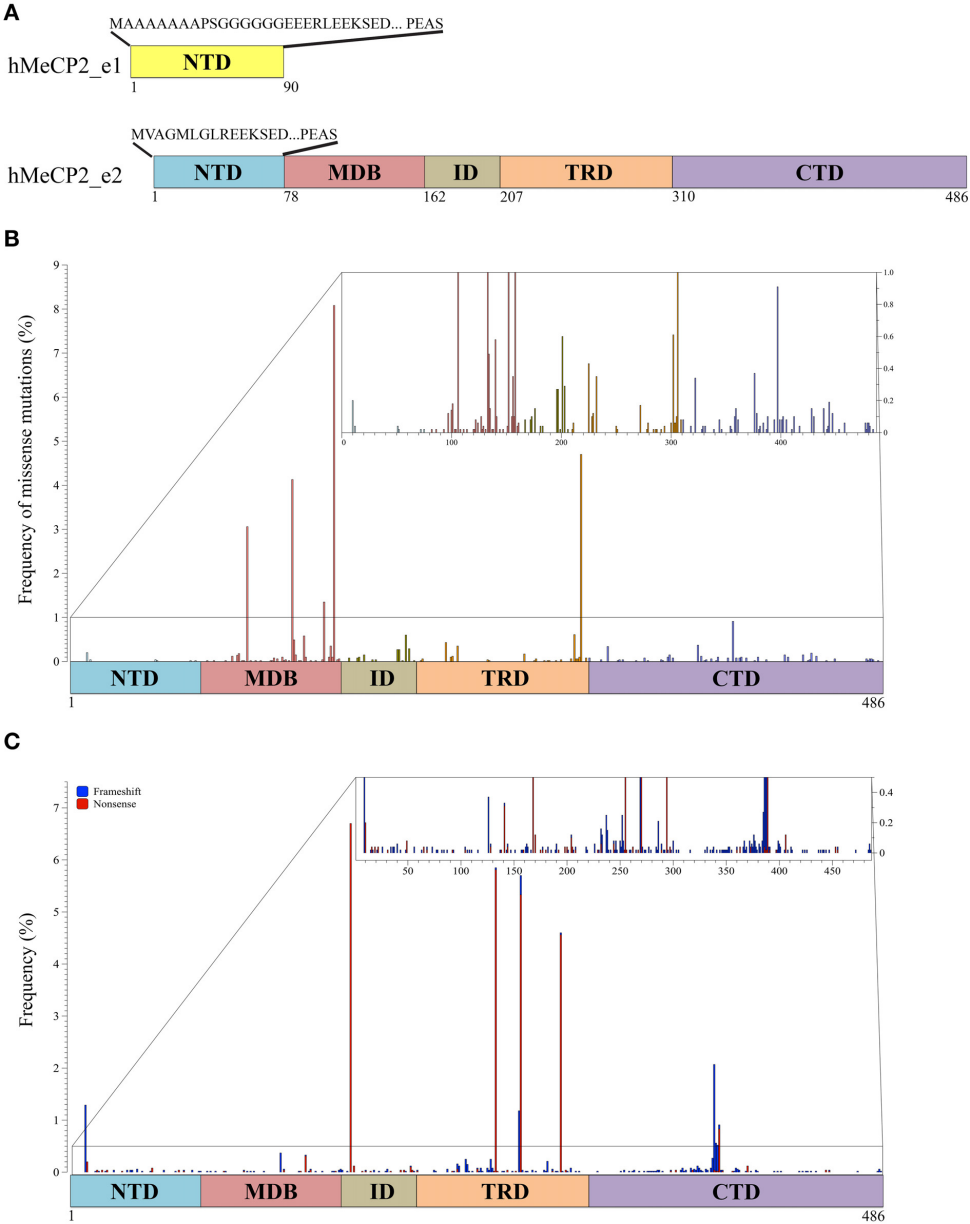
**MeCP2 isoforms and pathogenic mutations**. **(A)** Two MeCP2 isoforms, MeCP2_e1 and MeCP2_e2, are generated by alternative splicing originating two distinct N-terminal regions. MeCP2_e1 is 498 amino acids long and contains a N-terminal domain (NTD, yellow) of 90 amino acids of which the first 21 are distinct, whereas MeCP2_e2, formed by 486 amino acids, has 9 unique amino acids in its NTD (blue). MeCP2 is constituted by five sub-domains: NTD, MBD (methyl-CpG binding domain), ID (intervening domain), TRD (transcriptional repression domain, CTD (C-terminal domain); below the MeCP2_e2 isoform is shown the amino acid numbers of the different domains. **(B)** Schematic illustration showing the localization and the frequency of pathogenic missense mutations within MeCP2. The small inset shows in details the mutation frequency between 0 and 1%. The colors of the vertical bars correspond to the color code of the distinct MeCP2 subdomains. **(C)** Localization and frequency of non-sense and truncating *MECP2* mutations. Frameshift mutations are shown in blue and non-sense mutations in red. The small inset shows in details the mutation frequency between 0 and 1%.

MeCP2 has originally been described as an abundant and ubiquitously expressed nuclear protein that binds selectively methylated DNA (Lewis et al., 1992). In this first work, the primary structure of the human protein was defined as a polypeptide 486 residues long, containing two main domains, namely, a methyl-CpG binding domain (MBD) and a transcription repression domain (TRD). Nowadays, we know that alternative splicing, occurring both in human and mouse, generates two main isoforms of the protein (MeCP2_e1 and MeCP2_e2; Figure 1A; Chahrour and Zoghbi, 2007). Considering that (i) the primary structure of MeCP2 is highly conserved among vertebrates, (ii) the two principal isoforms differ exclusively in the very first N-terminal residues, and (iii) RTT mutations generally refer to the MeCP2_e2 isoform, in this review, we will mainly refer to the human MeCP2_e2 isoform (486 residues; Figure 1A). However, it is important to recall that MeCP2_e1 is the predominant isoform in brain. Furthermore, since MeCP2 PTMs have been investigated almost exclusively in rodents, in the future, it will be important to address which of the identified PTMs are maintained in the human protein and whether human MeCP2 is characterized by additional events of modification.

**Figure 2:**
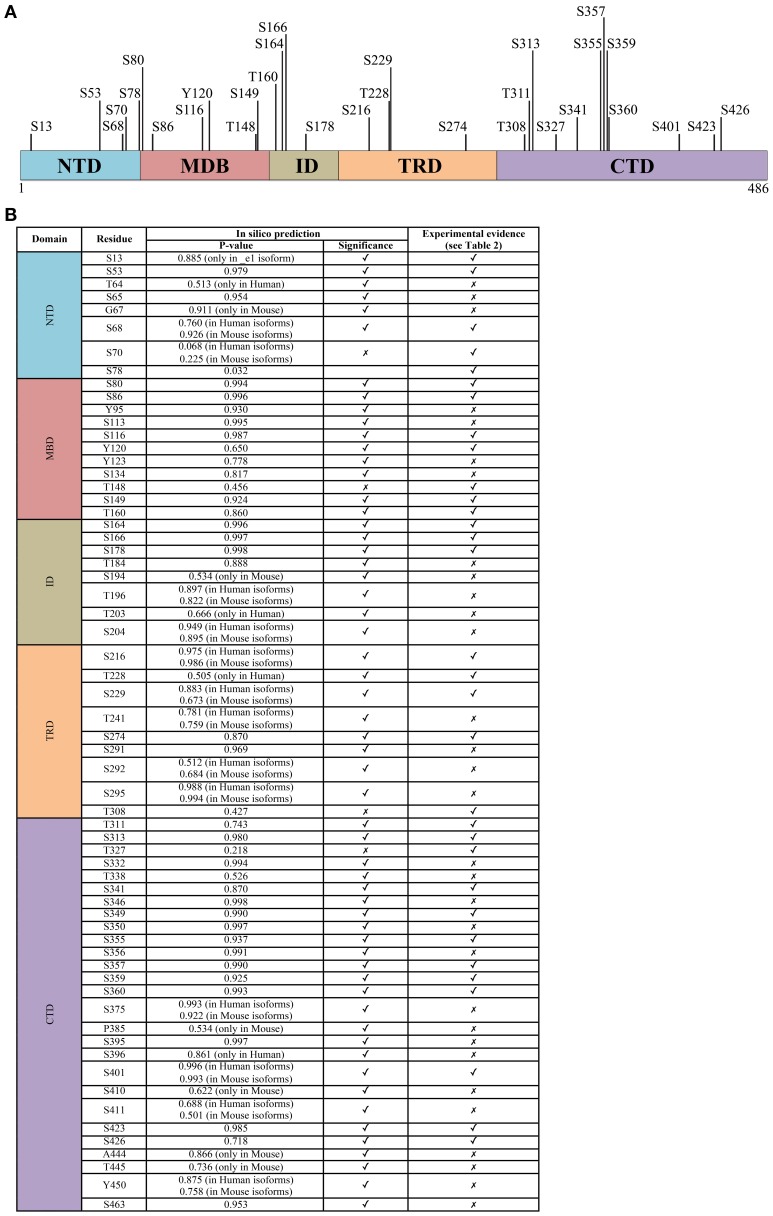
**The phosphorylation signature of MeCP2. (A)** All experimentally determined phosphorylation sites of MeCP2. The numbering corresponds to the hMeCP2_e2 isoform. **(B)** The probability score of phosphorylation sites within MeCP2 identified *in silico* by GPs 2.0 and NetPhos 2.0 confronted with experimentally determined sites; only residues identified as phosphorylation sites *in silico* with *P* > 0.5 are listed. Further details including references of experimentally determined sites are listed in supplementary Table 1.

**Figure 3:**
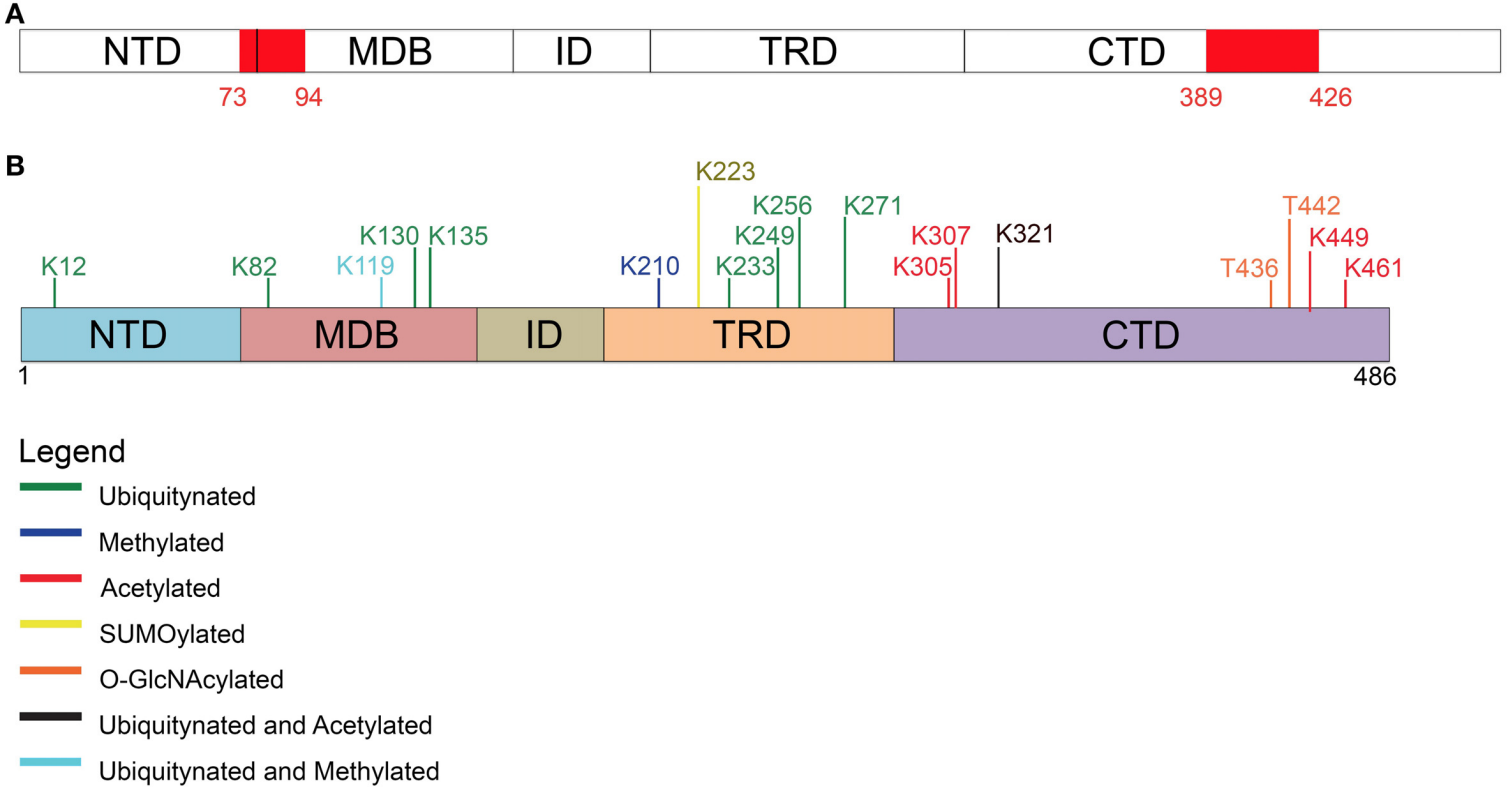
**Post-translational modifications other than phosphorylation affecting MeCP2**. **(A)** Two PEST motives, enriched in proline, glutamate, serine, and threonine residues, are present within MeCP2 possibly regulating its stability and phosphorylation. **(B)** Schematic representation of the different types of post-translational modifications (PTM) affecting MeCP2 and the involved amino acids.

As of today, MeCP2 has been subdivided into five main structural domains corresponding to the N-terminal domain (NTD), the MBD, the intervening domain (ID), the TRD and the C-terminal domain (CTD; Figure 1A). The MBD was originally defined as the minimum continuous ensemble of MeCP2 residues necessary and sufficient for selective binding to methylated CpG-dinucleotides (Nan et al., 1997). Its relevance for MeCP2 functions is highlighted by the fact that almost half of the known disease-causing missense mutations in *MECP2* occurs within this domain, including three of the eight most frequent RTT mutations (Figure 1B; for a complete updated database of *MECP2* mutations see www.Mecp2.chw.edu.au). *In vitro* studies have confirmed the relevance of MeCP2 binding to methylated DNA, suggesting that most, if not all, missense mutations within the methyl-binding domain impair the selectivity of MeCP2 for methylated DNA (Yusufzai and Wolffe, 2000). The X-ray structure of the MBD alone or associated with methylated DNA unexpectedly revealed that the MBD recognizes the hydration of the major groove, in which methylated DNA resides, rather than cytosine methylation *per se* (Ho et al., 2008). Importantly, these studies have been extended by the recent demonstration that the MBD of MeCP2 avidly binds both 5-methyl cytosine (5mC) and 5-hydroxymethyl cytosine (5hmC). This property has not been observed for the other members of the MBD family (MBD1-4). Interestingly, the pathogenic R133C MeCP2 mutant retains most of its binding to methylated DNA, but has lost affinity for 5hmC. Since small changes in the MBD structure seemed to influence the DNA binding properties of MeCP2, the authors proposed that PTMs affecting MeCP2 might alter its substrate specificity and, thus, its downstream functions (Mellen et al., 2012). In possible accordance with this hypothesis, the MBD has been found to be phosphorylated, methylated and acetylated (see more ahead and Figures 2, 3 and Supplementary Table 1). Interestingly, two lysine residues (K130 and K135) surrounding R133 have been found to be ubiquitinated (Gonzales et al., 2012). Although we do not yet know whether these residues are mono- or polyubiquitinated, several pieces of evidence prove that ubiquitin is a bulky modifier with possible direct steric effects on any modified protein, influencing conformational flexibility and protein-protein interactions. These studies thus suggest that ubiquitination might be a mechanism for a direct and rapid control of MeCP2 activities and interactions (Chernorudskiy and Gainullin, 2013). In the future, it would be interesting to address whether acetylated K130 and K133 affect the binding of MeCP2 to modified DNA.

The TRD was originally defined as the smallest region of MeCP2 required for transcriptional repression in functional assays (Lewis et al., 1992). No structural information is available for this domain or for the other remaining parts of MeCP2. The lack of structural information can easily be explained by Circular Dichroism studies and theoretical predictions demonstrating that almost 60% of MeCP2 is unstructured (Adams et al., 2007). Thus, MeCP2 is now recognized as an “intrinsically disordered protein” that might acquire local secondary structures upon binding to other macromolecules. Accordingly, MeCP2 is capable of multiple protein–protein interactions (Ausió et al., 2014; Bedogni et al., 2014) and appears to enter into a stable association with its cofactors only when bound to DNA. Thus, MeCP2 seems to act as a multifunctional factor that sustains interactions with specific partners depending on the architecture of its target DNA sequences (Klose and Bird, 2004). Recent findings, which will be discussed in details further on, suggest that also MeCP2 PTMs affect its protein-protein interactions. It is relevant that several of the modifications identified so far cluster in the transcriptional repression domain (Figure 2).

Protease digestion has been used to define the remaining NTD, ID and CTD domains (Adams et al., 2007); importantly, by analyzing the MeCP2 mutation database, we found that almost 25% of residues in the CTD and ID have been associated with pathogenic missense mutations, therefore suggesting their relevance for MeCP2 functions (Figure 1B; Bedogni et al., 2014). Although these two domains maintain a highly disorganized structure, the ID domain has been involved in several protein-protein interactions and diverse phosphorylation events (Bedogni et al., 2014 and Figure 2). On the contrary, to the best of our knowledge, the CTD has been found associated exclusively with Sdccag1, a mediator of nuclear export (Long et al., 2011). However, this domain, which is crucial for the binding of MeCP2 to chromatin, is subject to various modifications (Nikitina et al., 2007; Bedogni et al., 2014).

To summarize we can state that: (i) the highly disorganized structure of MeCP2 endows the protein with multiple functions; (ii) the structured MBD gives the protein its unique capability of interacting with both methylated and hydroxymethylated DNA; (iii) PTMs of MeCP2 are likely to affect directly its binding to DNA and protein partners, therefore contributing to the versatility of MeCP2.

## MeCP2: the complexity of a fundamental multifunctional protein

Adrian Bird and collaborators originally identified MeCP2 as a nuclear factor capable of binding DNA with at least one symmetrically methylated CpG-dinucleotide (Lewis et al., 1992) and repressing transcription mainly through its TRD (Nan et al., 1997). In this pioneering work the authors proposed that the extent of MeCP2 repression depends on its abundance in the nucleus, and that MeCP2 is capable of displacing histone H1 from chromatin to access its binding sites (Nan et al., 1997). A subsequent work demonstrated that MeCP2 uses its CTD to stabilize and asymmetrically protect linker DNA, thus mimicking the association of histone H1 with chromatin (Chandler et al., 1999). The capability of MeCP2 to play the role of an architectural chromatin protein was further supported by a report showing that MeCP2 is a potent chromatin-condensing factor, functioning directly without other corepressor or enzymatic activities (Georgel et al., 2003). The authors demonstrated that depending on its molar ratio to nucleosomes, MeCP2 assembles novel secondary and tertiary chromatin structures regardless of DNA methylation; these effects on large scale chromatin organization represent a good explanation for its ability to repress *in vivo* transcription at a distance.

A further link between the repressive activity of MeCP2 and chromatin compaction was established by demonstrating that the TRD binds to corepressor complexes (Sin3A and NCoR) with histone deacetylase activities, and that transcriptional repression of methylated DNA *in vivo* is partially relieved by the deacetylase inhibitor trichostatin A (Jones et al., 1998; Georgel et al., 2003). Importantly, through the years other chromatin-related partners of MeCP2 have been identified, such as Brahma, ATRX, CoREST, c-Ski, and H3K9 histone methyltransferase (Nan et al., 1998; Chahrour and Zoghbi, 2007; Bedogni et al., 2014).

All these results fit very well with more recent results demonstrating that in mature neurons, where MeCP2 abundance corresponds roughly to one molecule every second nucleosome, the protein is genome-wide bound, tracks methylated DNA, serves as an alternative linker histone, and organizes a specialized chromatin structure, thus dampening overall transcriptional noise (Skene et al., 2010; Guy et al., 2011). The effect on global genomic architecture is outlined by a selective increase in histone acetylation, H1 levels and transcription of repetitive elements and L1 retrotransposons in *Mecp2*-null neurons, but not in glia, where the protein is much less abundant (Moutri et al., 2010; Skene et al., 2010). Thus, the lack of functional MeCP2 in mature neurons might lead to a disorganized chromatin structure that impairs synaptic plasticity by preventing proper neuronal responses to stimuli. Cohen et al. (2011) have then recently reinforced the possibility that MeCP2 does not regulate the expression of specific genes, but rather functions as a histone-like factor, globally bound across the genome.

MeCP2 functions have been further expanded in the last years and a role in facilitating gene expression has also been suggested. In particular, the analysis of gene expression patterns in the hypothalamus of mice that either lack or overexpress Mecp2, led Huda Zoghbi and her colleagues to propose that MeCP2 predominantly activates transcription (Chahrour et al., 2008). The authors suggested that the association of MeCP2 with the transcriptional activator CREB1 might explain these results. Similarly, in a recent paper Rudolf Jaenisch and his collaborators demonstrated that the reduced size of *Mecp2*-null neurons affects total RNA levels per cell. By analyzing global transcriptional profiles starting from an identical number of cells, thus avoiding the usual normalization to total input RNA (Li et al., 2013), they found more down-regulated than up-regulated genes, leading to the conclusion that one of the key functions of MeCP2 is to facilitate global transcription. So far, the “per cell” perspective has been used only in one report (Li et al., 2013) and we still need to understand whether the discrepancy between the studies can be justified by the different approaches. Further, we suggest, as described in a recent publication, that higher-level bioinformatics, identifying statistically significant deregulated molecular pathways (rather than single genes), might allow the identification of the biological processes affected by dysfunctional MeCP2 (Bedogni et al., 2014).

In addition to all of this, it is important to recall that MeCP2 has also been proposed to play a role in regulating mRNA splicing and in modulating protein synthesis. In fact, Mecp2 was first found to interact with the RNA-binding protein Y box-binding protein 1 (YB1) and to regulate splicing of reporter minigenes. Supporting these data *in vivo*, aberrant alternative splicing patterns were also observed in a mouse model of RTT (Young et al., 2006; Maunakea et al., 2013). The involvement of MeCP2 in mRNA biogenesis has been further proven by the interaction of Mecp2 with Prpf3 (pre-mRNA processing factor 3), a spliceosome associated protein (Long et al., 2011). Eventually, a severe defect in protein synthesis in *Mecp2* mutant mice was for the first time demonstrated by Ricciardi et al. (2011). This deficiency, which is a common defect in autism spectrum disorders, was ascribed to a dysfunctional AKT/mTOR pathway. The relevance of these findings has subsequently been strengthened by the demonstration that pharmacological or genetic enhancement of protein synthesis ameliorates *Mecp2*-defective neurons (Li et al., 2013).

Thus, considering the structural flexibility of MeCP2 and the possibility of PTMs affecting its structure and/or interactions with protein partners, we believe that a better comprehension of MeCP2 functions might be obtained through the study of its diversely modified isoforms and their networking capabilities. Therefore, we will progress critically surveying the actual knowledge on MeCP2 phosphorylation that so far certainly represents the best characterized PTM of the methyl-binding protein.

## MeCP2 phosphorylation: where we stand and where we might go

The history of MeCP2 phosphorylation (pMeCP2) began in 2003 when two independent studies demonstrated that neuronal activation triggers a calcium-dependent phosphorylation of Mecp2 and the consequent release of the methyl-binding protein from *Bdnf* promoter III, thereby facilitating transcription (Chen et al., 2003; Martinowich et al., 2003). Several were the breaking news of these publications although some of them still remain debated.

To begin with, these studies proposed for the first time that Mecp2 is involved in the control of neuronal activity-dependent gene regulation through its PTMs, and that the deregulation of this process might participate in the RTT pathology. As we will see, this concept still remains valid and has been further strengthened by the development of Mecp2 phospho-defective transgenic mice (Table 1). Furthermore, these findings suggested that Mecp2 might work as a dynamic and selective regulator of neuronal gene expression.

**Table 1:** **Phosphorylation-defective *Mecp2* mouse models**.

	**Animal phenotype**	**Synaptic physiology**	**Molecular Phenotype**	**Importance**
***Mecp2^S80A^***; Tao et al., 2009	Normal lifespan;		Decreased binding to chromatin on specific gene promoters.	S80 phosphorylation regulates MeCP2 function in resting neurons.
	RTT-like phenotype;			
	−62% locomotor activity in dark cycle running wheel assays.			
***Mecp2^T308A^***; Ebert et al., 2013	Normal body weight;		T308A mutation does not alter MeCP2 stability, binding to DNA and basal interaction with NCoR complex;	Loss of phosphorylation-dependent interaction of MeCP2 with NCoR contributes to the development of some neurological defects observed in RTT.
	Reduced brain weight;			
	More seizures and lower seizure threshold;			
	Locomotor defects.		Decrease in membrane depolarization-induced *Npas4* and *Bdnf* expression.	
***Mecp2^S421A^***; Cohen et al., 2011	No visible differences compared to their wild type littermates;	Increased dendritic complexity;	No detectable effect on gene transcription.	S421 phosphorylation has a role in synaptic connections development within the cerebral cortex.
	No locomotor defects;	Increased mIPSC amplitude.		
	Behavioral abnormalities outlined with sociability and preference for social novelty assays.			
***Mecp2^S421A;S424A^***; Tao et al., 2009; Li et al., 2011	Normal lifespan;	Stronger LTP induced at both the Schaffer collateral-CA1 synapse and the mossy fiber-CA3 synapse, compared to wild type;	Hippocampal *Mecp2^S421A;S424A^* neurons show an increase in *Bmp4* and *Bdnf* transcription and a decrease in *Mef2c* and *Grm1* transcription.	S421 phosphorylation is a neuronal-activity induced event.
	No RTT-like phenotype;			
	+145% locomotor activity in dark cycle running wheel assays;			
	Fear-conditioning test and Morris water maze test suggest an altered hippocampus function.	Increased excitatory synaptogenesis in both the hippocampal and cortical neurons.		

The residue involved was then identified as serine 421 (S421 refers to mouse Mecp2_e2 and corresponds to S438 in Mecp2_e1 and S423 in hMeCP2_e2; see Figure 2); importantly, S421 phosphorylation occurs selectively in neural tissues and involves on the average 10–30% of the overall Mecp2 molecules (Zhou et al., 2006). By expressing a S421A phospho-defective derivative of Mecp2 in primary neurons, the authors demonstrated that this PTM affects dendritic growth, whereas the importance of S421 phosphorylation for circuit development was suggested by a defective patterning of distal apical dendrites in the brain of a phospho-defective S421A knock-in (KI) mouse line (*Mecp2^S421A/y^*, Cohen et al., 2011). Moreover, a shift in excitation-inhibition balance in favor of inhibition in Mecp2 S421A cortical slices was measured (Table 1). The relevance of S421 phosphorylation is emphasized by the fact that a similar shift has already been described in *Mecp2* knock-out mice. However, and possibly in accordance with the mild phenotype of the KI mouse that appears to be defective only in the capability of processing novel experience, so far no pathogenic mutation has been associated with this residue. Of relevance, we still lack insights regarding the molecular consequences of this PTM. As already mentioned, initial data suggested that S421 phosphorylation induces the detachment from specific genes. However, a ChIP-seq approach used to describe the genomic distribution of this specific Mecp2 phospho-isoform in brain of knock-in mice did not reveal a selective detachment of pMecp2 either in resting or stimulated conditions. These results might indicate that Mecp2 affects activity-dependent transcription by changing the molecular partners recruited on DNA (Chen et al., 2003).

By merging clinical data with those obtained from the S421A KI mice, we speculate that S421 phosphorylation regulates only some aspects of cognitive function, and that its deregulation leads only to subtle cognitive impairments. Accordingly, the analysis of the gene expression profiles did not reveal significant changes in the expression of individual genes. However, a possible mild effect on molecular pathways has not yet been addressed.

Importantly, S421 phosphorylation has also been associated with drug sensitivity and mood regulation. In fact, the research of stimuli affecting MeCP2 phosphorylation has led not only to demonstrate that hippocampus-dependent behavioral training leads to a robust increase in Mecp2 S421 phosphorylation (Li et al., 2011), but also that a single acute injection of cocaine or amphetamine elicits a transient increase in pS421 Mecp2 in the caudate putamen and nucleus accumbens (Deng et al., 2010; Mao et al., 2011). Having hypothesized that cellular and behavioral adaptations to these drugs might be affected by pS421, the authors have very recently produced the first data demonstrating that phosphorylation of MeCP2 at Ser421 functionally limits cellular sensitivity and synaptic response to repeated psychostimulant exposure in the mesocorticolimbic circuitry (Deng et al., 2014). A role for MeCP2 phosphorylation in the context of chronic opioid consumption and withdrawal has also been suggested: in fact, morphine withdrawal induces pS421Mecp2 in selected brain areas, such as lateral septum and the nucleus accumbens shell (Ciccarelli et al., 2013). Eventually, S421 phosphorylation in the nucleus accumbens and lateral habenula has been associated with depressive-like behaviors: in fact, administration of the antidepressant imipramine induces pS421Mecp2 and studies with knock-in mice showed that this induction is required for a proper response to the antidepressant (Hutchinson et al., 2012).

Mass spectrometry (MS) analysis identified that neuronal activity induces also S424 phosphorylation in mouse brain (isoform 2; Tao et al., 2009). A KI mouse line, in which the phosphorylation at both residues of the endogenous protein is abolished, was also generated (*Mecp2^S421A;S424A/y^*; Li et al., 2011). Before reviewing the results obtained, it is worth mentioning that MS failed to identify S424 phosphorylation in rat brain, therefore questioning the possible involvement of this PTM in RTT. At the molecular level, these two mutations did not affect the expression and intracellular localization of Mecp2; phenotypically, the obtained mice did not show any overt difference with respect to the wild-type animals. By exploiting conventional behavioral tests, the authors found a higher performance for some hippocampal functions, such as spatial memory, than in wild-type littermates. In accordance with enhanced hippocampus-dependent learning and memory, LTP was found significantly stronger in *Mecp2^S421A;S424A/y^* hippocampal slices with respect to the controls, together with an increase in excitatory synaptogenesis in both cortical and hippocampal cultured neurons. ChIP analyses permitted to hypothesize that the concomitant loss of these two phosphorylation sites enhances the binding of Mecp2 to its target gene promoters; importantly, the transcriptional outcome depends on the bound sequence, reinforcing the possibility that MeCP2 can function both as an activator and a repressor of transcription.

Summarizing the results obtained, both publications testify that MeCP2 phosphorylation impacts the development and function of the nervous system. Furthermore, they suggest that MeCP2 functions are regulated by several PTMs and their combination. Future studies need to address whether a crosstalk between MeCP2 PTMs exists, and moreover, the current contradiction concerning S424 phosphorylation needs to be solved. In fact, Michael Greenberg reported as unpublished results that they were unable to detect increased phosphorylation of Mecp2 S424 in response to neural activity both *in vitro* and *in vivo* (Cohen et al., 2011). Thus, it is unclear whether the S424A mutation in knock-in mice affects a phosphorylation site of Mecp2 or influences the molecular properties of the protein through an alternative mechanism. Although the production of a single *Mecp2^S424A/y^* KI line might be informative, we are not confident it would be convenient, also considering that so far this residue has never been associated with RTT.

By surveying MeCP2 phosphorylation in rat/mouse brains and in human HeLa cells, Tao et al. (2009) found that S80 phosphorylation is the most conserved phosphorylation site of MeCP2. S80 appears as the most abundantly phosphorylated residue under resting conditions whereas neuronal activity induces its dephosphorylation. *Mecp2^S80A/y^* knock-in mice are slightly overweight and show decreased locomotor activity (Tao et al., 2009). Functionally, S80 phosphorylation does not affect the overall subcellular localization of MeCP2, but seems to increase its affinity for chromatin. However, these *in vitro* studies, together with microarray analyses, were performed with exogenously expressed MeCP2 derivatives, expressed at levels slightly above those of the endogenous protein. Although no further data were produced with this *Mecp2* transgenic line, available information lead us to suggest that S80 phosphorylation does not have any impact on global transcription and that, probably, this MeCP2 phospho-isoform is globally bound to chromatin, therefore resembling the distribution of MeCP2 and the pS421 isoform.

A more recent work used phospho-tryptic maps to confirm that Mecp2 is phosphorylated at many sites in cultured activated and resting neurons (Ebert et al., 2013). In this study, the activity-dependent phosphorylation of Mecp2_e2 occurs on residues S86, S274, T308, and S421 (for an easier comprehension of the state of art, all the so-far-identified MeCP2 phospho-sites are summarized in Figure 2 and Supplementary Table 1). Considering that the nearby R306 residue is mutated in RTT, and that its pathogenic R306C mutation disrupts the ability of MeCP2 to interact with the corepressor complex NCoR (Lyst et al., 2013), the authors focused their attention on T308 phosphorylation and found that it abolishes the interaction of MeCP2 with the corepressor, thereby reducing MeCP2-mediated transcriptional repression. The phospho-defective *Mecp2^T308A/y^* KI mice demonstrated that mutant MeCP2 maintains its global distribution on chromatin, but that activity dependent induction of MeCP2 target genes is deficient. Importantly, the brains of these mice weigh significantly less, the animals display motor system defects, and have a lower seizure threshold compared to wild-type mice. Since all these symptoms recapitulate the manifestation of several *Mecp2* mouse models, this is a groundbreaking piece of work highlighting not only the relevance of MeCP2 phosphorylation but also its role as a transcriptional silencer through its interaction with the corepressor complex NCoR/HDAC.

Eventually, the laboratory of Janine La Salle used SH-SY5Y cells stably expressing FLAG-tagged mouse MeCP2_e1 to identify MeCP2 PTMs by tandem mass spectrometry and, interestingly, found them to cluster mainly in the MBD and TRD domains (Gonzales et al., 2012). Regarding phosphorylation, six sites were identified, partly overlapping with previous data (Figure 2). The successful production of two phospho-specific antisera, against pS80 and pS229, permitted to confirm that these modifications are independent of one another, and can coexist on the same molecule of MeCP2 (Tao et al., 2009). Whereas both Mecp2 phospho-isoforms were found in brain and were characterized by the same subnuclear distribution as total Mecp2, pS229Mecp2 showed enriched binding to a tested promoter with respect to total Mecp2. Moreover, it was observed that pS80Mecp2 and pS229Mecp2 have a preferential association with distinct combinations of MeCP2 cofactors compared to total MeCP2, confirming the hypothesis that MeCP2 changes its partners and mechanisms of action through its PTMs. Accordingly, the phosphorylation of S80 seems to affect the interaction of MeCP2 with RNA and the RNA-binding protein YB-1 (Gonzales et al., 2012).

Summarizing, most data suggest that MeCP2 is a multifunctional protein that “transiently” performs its function(s) depending on its differential phosphorylation. Accordingly, although S421 phosphorylation remains for the time being the most widely characterized PTM and pT308 results as the most functionally relevant, several other sites of phosphorylation have been mapped, and their modification is often spatially regulated by specific stimuli. Almost no information is available so far on these PTMs and their regulation during brain development; furthermore, since a role of MeCP2 outside of the nervous system, particularly in glia and microglia, has recently been demonstrated as important for MECP2-related disorders, future studies should also investigate MeCP2 phosphorylation in these cells. MeCP2 phosphorylation has been hypothesized to affect its binding to target DNA sequences: however, no data seem to support this hypothesis in neurons, and none of the phosphorylation events characterized so far globally affect MeCP2 binding to chromatin. It is highly plausible then that the identification and/or characterization of novel phospho-residues of MeCP2 will lead to the discovery of PTMs that generally or selectively influence its binding to chromatin. Furthermore, they should help providing a better comprehension of MeCP2 in physiological and pathological conditions. To this purpose, we suggest that studies should focus first on sites that are conserved by evolution and/or have been found mutated in patients. Thus, we employed two different tools to predict phosphorylation sites (GPs 2.0, Xue et al., 2008; and NetPhos 2.0, Blom et al., 1999), at the same time screening the existent proteomic literature to reveal which residues have already been found modified in cell lines or *in vivo*. The results are summarized in Figure 2. Algorithms for the prediction of PTMs in proteins are usually based on machine learning approaches, and associate with the predictions a reliability score ranking the residues according to their likelihood of being modified. The two algorithms we employed yielded highly consistent results. All in all, the strong correlation between experimentally verified phosphorylated sites and their ranking in the predictions leads us to conjecture that sites with high prediction scores but not yet validated, usually considered as “false positives,” might indeed be very likely candidates for phosphorylation, and worth of further experimental investigation.

Eventually, a thorough characterization of MeCP2 phosphorylation should also address which are the signaling pathways involved, and the respective kinases and phosphatases. No phosphatases have been described yet: on the contrary, a few studies (Chen et al., 2003; Martinowich et al., 2003; Zhou et al., 2006; Bracaglia et al., 2009; Tao et al., 2009; Khoshnan and Patterson, 2012; Ciccarelli et al., 2013) have suggested kinases that either directly or indirectly affect MeCP2 phosphorylation. Since the data obtained so far do not appear as conclusive, and we consider bioinformatics approaches not yet reliable enough to obtain informative data on this aspect (data not shown), we have decided not to address further this issue in this work.

## MeCP2 post-translational modifications other than phosphorylation and conclusions

The heterogeneity of MeCP2 posttranslational modifications, together with its genome-wide distribution (Skene et al., 2010), its capacity to mediate the formation of a highly compacted chromatin structure *in vitro* (Georgel et al., 2003) and to substitute histone H1 *in vivo* (Skene et al., 2010) have led some authors to suggest that, when highly abundant, the protein might behave as a specialized histone-like chromatin organizing factor. Thus, in analogy with the histone code, it is highly conceivable, as already hinted along this review, that MeCP2 PTMs affect its binding to specific epigenetic marks as well as its interactions with protein partners and therefore the resulting biological functions. In accordance with this hypothesis, there are a few studies demonstrating the existence on MeCP2 of PTMs different from phosphorylation on MeCP2. Thus, in this last section we will briefly survey the information existing so far. Available data are still very limited, no animal models have been generated and, often, functional studies have not been performed in neurons. Future studies will certainly have to fill this gap of knowledge.

In detail, Gonzales et al. (2012) used SH-SY5Y cells stably expressing epitope-tagged MeCP2_e1 to analyze its PTMs by tandem mass spectrometry with 90% coverage. Importantly, apart from the already mentioned phosphorylation events, 10 ubiquitination sites were identified (Figure 3 and Table 2), with lysine 271 occurring as the most frequently modified site. Although, no functional role has been yet provided for these PTMs, all of them but two reside in the MBD or TRD domains leading to the hypothesis that they might affect the binding of MeCP2 to chromatin or its regulatory properties. These studies have been performed in cultured cell lines and with exogenously expressed MeCP2: however, their relevance might be underlined by the fact that three of these sites have been associated to missense mutations in RTT (Table 2). Eventually, by surveying literature we found that lysine 130 had already been found ubiquitinated (Wagner et al., 2011). The functional significance of ubiquitination is dual: generally, it is associated with the rapid degradation of modified proteins; however, such modification can also have a role in signaling or trafficking. Interestingly, Ausiò and his collaborators have identified two strong, conserved PEST motives in the primary sequence of MeCP2 (Figure 3). PEST motives are sequences enriched in proline, glutamate, serine, and threonine residues, and often predispose proteins to rapid proteolytic degradation (Thambirajah et al., 2009). The PEST structure gets destabilized upon phosphorylation leading to the degradation of the modified protein. Interestingly, although no connection to protein stability has been established so far, the two best-defined phosphorylation events (S80 and S421) reside in the PEST domains. In addition to phosphorylation, authors have identified several potential ubiquitination sites at lysine residues flanking the PEST domains. The hypothesis that the two PEST domains have a major role in fine-tuning MeCP2 levels and that PTMs associated with the modification of these domains regulate the activity of MeCP2 is a challenging one. However, no further data have been provided to support it and evidence for the ubiquitination of these residues is still lacking.

**Table 2:** **Post-translational modifications within MeCP2 other than phosphorylation**.

**Residue**	**Mutation in RTT**	**PTM**	**Cell line/Tissue assayed**	**References**
K12	K12N (0.04%)	Ub	SH-SY5Y neuroblast. cells	Gonzales et al., 2012
K82	K82fs (0.02%) K82R (0.02%)	Ub	SH-SY5Y neuroblast. cells	Gonzales et al., 2012
K119	–	Ub	SH-SY5Y neuroblast. cells	Gonzales et al., 2012
	–	Met	293 T	Jung et al., 2008
K130	–	Ub	HEK-293T SH-SY5Y neuroblast. cells	Wagner et al., 2011; Gonzales et al., 2012
K135	K135E (0.15%)	Ub	SH-SY5Y neuroblast. cells	Gonzales et al., 2012
R162	R162fs (0.06%)	Met	Kidney (7945, 7947)	www.PhosphoSite.org (Hornbeck et al., 2012), CST curation sets 7945, 7946, 7947, 7948, (Guo et al., 2014)
			Lung (7946, 7948)	
			Mice brain	
K210	K210I (0.02%)	Met	293 T	Jung et al., 2008
K223	K223NfsX3 (0.02%)	SUMO	Primary cortical neurons	Cheng et al., 2014
K233	K233fs (0.02%)	Ub	SH-SY5Y neuroblast. cells	Gonzales et al., 2012
K249	–	Ub	SH-SY5Y neuroblast. cells	Gonzales et al., 2012
K256	K256SfsX17 (0.02%)	Ub	SH-SY5Y neuroblast. cells	Gonzales et al., 2012
K271	–	Ub	SH-SY5Y neuroblast. cells	Gonzales et al., 2012
K305	K305fs (0.02%) K305E (0.02%) K305R (0.08%)	Ac	SH-SY5Y neuroblast. cells	Gonzales et al., 2012
K307	–	Ac	SH-SY5Y neuroblast. cells	Gonzales et al., 2012
K321	K321Sfs^*^13 (0.02%)	Ac	SH-SY5Y neuroblast. cells	Gonzales et al., 2012
		Ub	SH-SY5Y neuroblast. cells	Gonzales et al., 2012
T436	–	O-GlcNAc	Murine synaptosomes (Trinidad et al., 2012)	Wang et al., 2010; Trinidad et al., 2012
			Rat brain (Wang et al., 2010)	
T442	T442fs (0.02%)	O-GlcNAc	Rat brain	Wang et al., 2010
	T442A (0.02%)			
K449	–	Ac	MV4-11 (Choudhary et al., 2009)	(Choudhary et al., 2009)
			HCT 116 (CST)	www.PhosphoSite.org (Hornbeck et al., 2012), CST curation sets 9796, 9797
K461	–	Ac	HEK-293 cells and primary cortical neurons	(Zocchi and Sassone-Corsi, 2012)

More recently, MeCP2 has been found modified by the covalent linkage of small ubiquitin-like modifier (SUMO) to several lysines. In particular, the sumoylation of lysine 223 is required for the recruitment of HDAC1/2 complexes and mutation of K223 abolishes its gene silencing properties in primary cortical neurons (Cheng et al., 2014). Furthermore, this mutation impacts proper excitatory synaptogenesis *in vitro* and *in vivo* suggesting the relevance of sumoylation for MeCP2 functions.

In addition, Mecp2 has been found acetylated in several residues (Choudhary et al., 2009; Gonzales et al., 2012; Zocchi and Sassone-Corsi, 2012); in particular, acetylation of K464 of Mecp2_e1 has been identified in cultured cortical neurons. The authors have demonstrated that this modification is mediated by p300 and erased by the nicotinamide-adenine dinucleotide dependent histone deacetylase SIRT1. Chromatin immunoprecipitation experiments performed in *Sirt1*-null mice have led to hypothesize that K464 acetylation affects Mecp2 binding to DNA. However, in this work only the BDNF promoter has been tested.

Finally, MeCP2 has been found methylated in 293T cells (Jung et al., 2008) and O-glycosylated in 293T cells and rat brain (Rexach et al., 2010). For convenience, we provide a table summarizing the residues of MeCP2 that have been found involved in PTMs different from phosphorylation (SI Table 2). In this table, as well as in SI Table 1, we show the cell line/tissue in which the PTM was identified, the corresponding references, and the possible association of the residue involved with Rett syndrome. Few modified residues of MeCP2 have been linked to pathological conditions and with a very low incidence: thus, so far it is impossible to draw any genotype-phenotype correlation.

To conclude, the studies reviewed strengthen the hypothesis that a complex combinatorial pattern of PTMs functions as a regulatory platform of the methyl-binding protein. Once again the RTT field is awaiting the discovery of the functional consequences of these PTMs and their combinations. Their role in regulating synaptic plasticity is of special interest since deficits in MeCP2 has a strong impact on synaptic functions. Finally, the identification of their possible “readers,” as well as of effecting enzymes (“writers”) and their possible involvement in MECP2 related disorders represent urgent challenges.

## Author contributions

Nicoletta Landsberger wrote the manuscript with the assistance of Charlotte Kilstrup-Nielsen; Elisa Bellini generated the figures and the tables; Giulio Pavesi performed the *in silico* analyses; Isabella Barbiero, Anna Bergo, Chetan Chandola, Mohammad S. Nawaz, Laura Rusconi, Gilda Stefanelli, Marta Strollo, and Maria M. Valente contributed equally in the conception and drafting of the manuscript.

### Conflict of interest statement

The authors declare that the research was conducted in the absence of any commercial or financial relationships that could be construed as a potential conflict of interest.

## Abbreviations

MeCP2: methyl-CpG binding protein 2
RTT: Rett syndrome
PTM: post-translational modification
MBD: methyl-DNA binding domain
TRD: transcriptional repression domain
ID: intervening domain
NTD: N-terminal domain
CTD: C-terminal domain
5hmC: 5-hydroxymethyl cytosine
pMeCP2: phosphorylated MeCP2
ChIP: chromatin immunoprecipitation
KI: knock-in
MS: mass spectrometry
LTP: long-term potentiation
Ub: ubiquitination
Met: methylation, SUMO, sumoylation
Ac: acetylation
O-GlcNAc: O-glycosylation.
