# Supplementary material for: MeCP2 post-translational modifications: a mechanism to control its involvement in synaptic plasticity and homeostasis?
Source: Front Cell Neurosci. 2014 Aug 13;8:236. doi: 10.3389/fncel.2014.00236 (PMC4131190; doi:10.3389/fncel.2014.00236)
Supplement: Supplementary file 1 [file DataSheet1.DOCX]

**SUPPLEMENTARY DATA Bellini et al., 2014**

**Supplementary Table 1. Summary of experimentally identified phosphorylation sites within MeCP2.**

Information of correspondence with pathogenic MECP2 mutations (mutation frequency shown in percentage) and the cell line/tissue in which the phosphorylation was identified are indicated together with the corresponding reference.

| **Residue** | **Mutation in RTT** | **Cell line/Tissue assayed** | **Ref.** |
| --- | --- | --- | --- |
| S13 | - | - SH-SY5Y neuroblastoma cells (Gonzales et al., 2012) - Colorectal cancer (CRC) samples (Shiromizu, 2013) - Jurkat cell line (CST) | Gonzales et al., 2012  Shiromizu et al., 2013  www.PhosphoSite.org (Hornbeck et al., 2012), CST curation set 12495 |
| S53 | - | Colorectal cancer (CRC) samples (Shiromizu, 2013) | Shiromizu et al., 2013 |
| S68 | - | Brain, brown fat, heart, liver, lung, kidney, pancreas, spleen, and testis of Swiss-Webster mice | Huttlin et al., 2010 |
| S70 | - | Brain, brown fat, heart, liver, lung, kidney, pancreas, spleen, and testis of Swiss-Webster mice | Huttlin et al., 2010 |
| S78 | S78fs (0.02%) | - HeLa cells (Dephoure et al., 2008; Olsen et al., 2010) - Mice brain tissue (Tweedie-Cullen et al., 2009) - Primary human leukocytes (Raijmakers et al., 2010) - mpkCCD cells (Rinschen et al, 2010) - Human embryonic stem cell and induced pluripotent stem cell lines (Phanstiel et al., 2011) - HeLa S3 cells (Olsen et al., 2010) - TG3 mutant mice melanoma (Zanivan et al., 2008) | Dephoure et al., 2008  Zanivan et al., 2008  Tweedie-Cullen et al., 2009  Olsen et al., 2010  Rinschen et al, 2010  Raijmakers et al., 2010  Yu et al., 2011  Phanstiel et al., 2011 |
| S80 | - | - HeLa cells (Beausoleil et al., 2004; Dephoure et al., 2008; Tao et al., 2009; Chen et al., 2009; Hsu et a., 2011; Zhou et al., 2013) - HEK-293E, HEK-293T and MEFs (Hsu et a., 2011) - MCF-7, and Du145 (Chen et al., 2009) - Human Jurkat T cell leukemia cell line (Mayya et al., 2009) - MEFs (Bracaglia et a., 2009) - 32Dcl3 (Choudhary et al., 2009) - PC12 cell (Tao et al., 2009) - Mice brain tissue (Tweedie-Cullen et al., 2009; Wiśniewski et al., 2010; Goswami et al., 2012) - Human brain tissue (Herskowitz et al., 2010) - Malignant breast cancer cells (Iliuk et al., 2010) - Primary human leukocytes (Raijmakers et al., 2010) - Primary human T lymphocytes (Carrascal et al., 2008) - Lung cancer cells (Wang et al., 2010) - Non-small-cell lung cancer cell lysate (Tsai et al., 2008) - TG3 mutant mice melanoma (Zanivan et al., 2008) - AT1R stable transfected HEK293 (AT1R-HEK) cells (Christensen et al., 2010) - EBV-transformed B-lymphocyte cells (Bennetzen et al., 2010) - HeLa S3 cells (Olsen et al., 2010) - hESC (Phanstiel et al., 2011; Rigbolt et al., 2011) - iPSC (Phanstiel et al., 2011) - SH-SY5Y neuroblastoma cells (Gonzales et al., 2012) - Murine liver mitochondria (Grimsrud et al., 2012) - KG1 AML cells (Weber et al., 2012) - Colorectal cancer (CRC) samples (Shiromizu, 2013) - K562 cell line (CST Curation Set 4390, 2008; Zhou et al., 2013) - IEC-6 (Courcelles et al., 2013) - Rat liver (Demirkan et al., 2012) - Rat neuronal cells (Zhou et al., 2006) | Beausoleil et al., 2004  Zhou et al., 2006  www.PhosphoSite.org (Horbneck et al., 2012), CST curation sets 4390  Carrascal et al., 2008  Dephoure et al., 2008  Zanivan et al., 2008  Bracaglia et al., 2009  Chen et al., 2009  Choudhary et al., 2009  Mayya et al., 2009  Tao et al., 2009  Tweedie-Cullen et al., 2009  Bennetzen et al., 2010  Christensen et al., 2010  Herskowitz et al., 2010  Iliuk et al., 2010  Olsen et al., 2010  Raijmakers et al., 2010  Wang et al., 2010  Wiśniewski et al., 2010  Hsu et a., 2011  Phanstiel et al., 2011  Rigbolt et al., 2011  Demirkan et al., 2012  Gonzales et al., 2012  Goswami et al., 2012  Grimsrud et al., 2012  Weber et al., 2012  Courcelles et al., 2013  Shiromizu et al., 2013  Zhou et al., 2013 |
| S86 | S86C (0.02%) | Primary neuronal cells | Ebert et al., 2013 |
| S116 | S116fs (0.02%) | HeLa cells | Dephoure et al., 2008 |
| Y120 | Y120D (0.02%) | HeLa cells | Dephoure et al., 2008 |
| T148 | - | PC12 cell | Tao et al., 2009 |
| S149 | - | - PC12 cell (Tao et al., 2009) - HeLa S3 cells (Olsen et al., 2010) | Tao et al., 2009  Olsen et al., 2010 |
| T160 | T160S | Mice brain tissue (Tweedie-Cullen et al., 2009) | Tweedie-Cullen et al., 2009 |
| S164 | - | - PC12 cell (Tao et al., 2009) - Mice brain tissue (Tweedie-Cullen et al., 2009) | Tao et al., 2009  Tweedie-Cullen et al., 2009 |
| S166 | G166fs (0.02%) | Brain, brown fat, heart, liver, lung, kidney, pancreas, spleen, and testis of Swiss-Webster mice | Huttlin et al., 2010 |
| S178 | - | - Brain tissue (CST 12738) - Colorectal cancer (CRC) samples (Shiromizu, 2013) | www.PhosphoSite.org (Horbneck et al., 2012), CST curation sets 12738  Shiromizu et al., 2013 |
| S216 | - | - PC12 cell (Tao et al., 2009) - HeLa S3 cells (Olsen et al., 2010) - Primary peritoneal macrophages (Wu et al., 2012) - Colorectal cancer (CRC) samples (Shiromizu, 2013) - IEC-6 (Courcelles et al., 2013) | Tao et al., 2009  Olsen et al. 2010  Wu et al., 2012  Courcelles et al., 2013  Shiromizu et al., 2013 |
| T228 | T228S (0.1%) | hESC (Rigbolt et al., 2011) | Rigbolt et al., 2011 |
| S229 | S229L (0.12%) | - PC12 cell (Tao et al., 2009) - 32Dcl3 (Choudhary et al., 2009) - Mice brain tissue (Tweedie-Cullen et al., 2009; Wiśniewski et al., 2010; Goswami et al., 2012) - hESC (Rigbolt et al., 2011) - SH-SY5Y neuroblastoma cells (Gonzales et al., 2012) - Colorectal cancer (CRC) samples (Shiromizu, 2013) - HeLa (Chen et al., 2009; Zhou et al., 2013) - K562 (Zhou et al., 2013) - HeLa, MCF-7, and Du145 (Chen et al., 2009) - Rat neuronal cells (Zhou et al., 2006) | Zhou et al., 2006  Chen et al., 2009  Tao et al., 2009  Choudhary et al., 2009  Tweedie-Cullen et al., 2009  Wiśniewski et al., 2010  Rigbolt et al., 2011  Gonzales et al., 2012  Goswami et al., 2012  Shiromizu et al., 2013  Zhou et al., 2013 |
| S274 | S274fs (0.02%) | - Mice brain tissue (Tweedie-Cullen et al., 2009) - SH-SY5Y neuroblastoma cells (Gonzales et al., 2012) - Primary neuronal cells (Ebert et al., 2013) - Colorectal cancer (CRC) samples (Shiromizu, 2013) | Tweedie-Cullen et al., 2009  Gonzales et al, 2012  Shiromizu et al., 2013  Ebert et al., 2013 |
| T308 | - | Primary neuronal cells | Ebert et al., 2013 |
| T311 | T311M | Brain, brown fat, heart, liver, lung, kidney, pancreas, spleen, and testis of Swiss-Webster mice | Huttlin et al., 2010 |
| S313 | - | Colorectal cancer (CRC) samples | Shiromizu et al., 2013 |
| T327 | - | Colorectal cancer (CRC) samples | Shiromizu et al., 2013 |
| S341 | S341fs (0.02%) | Rat neuronal cells | Zhou et al., 2006 |
| S355 | - | - NCI-H1703 (CST curation set 5014, 2008) - Jurkat cell line (CST Curation Set 3770, 2008) | www.PhosphoSite.org (Hornbeck et al., 2012), CST curation sets 5014, 3770, |
| S357 | S357del (0.02%) | - NCI-H1703 (CST curation set 5014, 2008) - Human skin fibroblast cells (Yang et al, 2006) | www.PhosphoSite.org (Hornbeck et al., 2012), CST curation set 5014  Yang et al., 2006 |
| S359 | S359P (0.15%) | Human skin fibroblast cells | Yang et al., 2006 |
| S360 | S360fs (0.02%) | - Human skin fibroblast cells (Yang et al, 2006) - Human embryonic stem cell and induced pluripotent stem cell lines (Phanstiel et al., 2011) - Murine liver mitochondria (Grimsrud et al., 2012) - Colorectal cancer (CRC) samples (Shiromizu, 2013) | Yang et al., 2006  Phanstiel et al., 2011  Grimsrud et al., 2012  Shiromizu et al., 2013 |
| S401 | S401N (0.08%)  S401fs (0.04%) | - PC12 cell (Tao et al., 2009) - SH-SY5Y neuroblastoma cells (Gonzales et al., 2012) | Tao et al., 2009  Gonsalez et al., 2012 |
| S423 | - | - Primary murine neuronal cell (Ebert et al., 2013) - PC12 cell (Tao et al., 2009) - Cortical neurons (Cohen et al., 2011) - Murine synaptosomes (Trinidad et al., 2012) - Colorectal cancer (CRC) samples (Shiromizu, 2013) - Mice brain tissue (Tweedie-Cullen et al., 2009; Deng et al., 2010) - Rat neuronal cells (Zhou et al., 2006) | Zhou et al., 2006  Tao et al., 2009  Tweedie-Cullen et al., 2009  Deng et al., 2010  Cohen et al., 2011  Trinidad et al., 2012  Ebert et al., 2013  Shiromizu et al., 2013 |
| S426 | S426fs (0.02%) | - HeLa cells (Dephoure et al., 2008) - PC12 cell (Tao et al., 2009) - Brain, brown fat, heart, liver, lung, kidney, pancreas, spleen, and testis of Swiss-Webster mice (Huttlin et al., 2010) | Dephoure et al., 2008  Tao et al., 2009  Huttlin et al., 2010 |
